# Patterns in Dried Droplets to Detect Unfolded BSA

**DOI:** 10.3390/s22031156

**Published:** 2022-02-03

**Authors:** Yojana J. P. Carreón, Mary Luz Gómez-López, Orlando Díaz-Hernández, Pamela Vazquez-Vergara, Rosario E. Moctezuma, José M. Saniger, Jorge González-Gutiérrez

**Affiliations:** 1 Instituto de Ciencias Aplicadas y Tecnología, Universidad Nacional Autónoma de México, Av. Universidad 3000, Coyoacan, Mexico City 04510, Mexico; jose.saniger@icat.unam.mx; 2 Facultad de Ciencias en Física y Matemáticas, Universidad Autónoma de Chiapas, Tuxtla Gutiérrez 29050, Mexico; mary.gomez@cinvestav.mx (M.L.G.-L.); orlando.diaz@unach.mx (O.D.-H.); 3 Departament de Física de la Materia Condensada, Universitat de Barcelona, Av. Diagonal 645, E08028 Barcelona, Spain; pvazquezfmc@ub.edu; 4 Instituto de Física “Manuel Sandoval Vallarta”, San Luis Potosí 78000, Mexico; rosario@ifisica.uaslp.mx

**Keywords:** patterns, dried droplets, folded proteins, unfolded proteins

## Abstract

The morphological analysis of patterns in dried droplets has allowed the generation of efficient techniques for the detection of molecules of medical interest. However, the effectiveness of this method to reveal the coexistence of macromolecules of the same species, but different conformational states, is still unknown. To address this problem, we present an experimental study on pattern formation in dried droplets of bovine serum albumin (BSA), in folded and unfolded conformational states, in saline solution (NaCl). Folded proteins produce a well-defined coffee ring and crystal patterns all over the dry droplet. Depending on the NaCl concentration, the crystals can be small, large, elongated, entangled, or dense. Optical microscopy reveals that the relative concentration of unfolded proteins determines the morphological characteristics of deposits. At a low relative concentration of unfolded proteins (above 2%), small amorphous aggregates emerge in the deposits, while at high concentrations (above 16%), the “eye-like pattern”, a large aggregate surrounded by a uniform coating, is produced. The radial intensity profile, the mean pixel intensity, and the entropy make it possible to characterize the patterns in dried droplets. We prove that it is possible to achieve 100% accuracy in identifying 4% of unfolded BSA contained in a protein solution.

## 1. Introduction

Proteins are the principal component in all living systems, responsible for diverse molecular functions in biological systems. The specific three-dimensional structure of proteins is necessary to maintain their biological function in the cell. However, in certain circumstances, the protein concentration, complex interaction with other proteins, and the cellular environment can lead to misfolding or the formations of aggregates of proteins [1]. In this process, the affected protein can lose its biological function, develop resistance to degradation, and may acquire toxicity [1,2,3]. Unfolded proteins interact inappropriately with other molecules and are often aggregated. Some diseases derived from an “incorrect” folding of a specific protein or protein complex are cystic fibrosis, Marfan syndrome, and amyotonic lateral sclerosis [4]. Moreover, the aggregation and accumulation of proteins in the form of amyloids and plaques are distinctive elements of over 20 degenerative diseases, which affect diverse peripheral tissues and the central nervous system [3]. For example, the accumulation of amyloids deposit on different parts of the body are related to systemic amyloidosis, such as AL amyloidosis (accumulation of immunoglobulin light chain amyloid fibrils), amyloid A (deposits of catabolic products of the SSA protein), and ATTR (transthyretin accumulation) [5]. Moreover, type 2 diabetes may be due to pancreatic accumulation of islet amyloid polypeptide, affecting a single organ [6,7]. Protein aggregation diseases, which affect and are toxic to the central nervous system, are Huntington’s disease, Parkinson’s disease, prion diseases, and Alzheimer’s disease [1,3,7]. Therefore, developing techniques for the detection of conformational changes in proteins contributes to the understanding of pathologies and improves their care.

Pattern analysis of dried droplets of biofluids is useful for obtaining information about the existence and the structural state of the components of a solution [8,9,10,11]. For example, a reduction in radial crack lengths in dried droplets of whole blood helps to detect Thalassaemia, a blood disorder where hemoglobin (Hb) is abnormal [12]. In addition, the length of the large cracks is related to the level of bilirubin in the blood of infants with jaundice [13]. The changes in the size and spatial distribution of crack and plaque patterns in stains of whole blood help to correlate anemia and hyperlipidemia in patients [14]. This occurs because of the number of red blood cells decreasing in anemia, while high levels of triglycerides and cholesterol in the blood appear in hyperlipidemia. On the other side, fern-like patterns formed by dried droplets of cervical mucus detect the preterm premature rupture of membranes and/or the onset of labor due to the presence of NaCl in mucus under estrogen effect [15]. Prominent structures containing randomly distributed voids can be detected when the bilayer of liposomes transforms from an ordered gel phase to a disordered fluid phase [16]. A ring-like structure, so-called coffee ring, can characterize the bacterial functionality due to motility during biological processes [17,18].

The occurrence of patterns in deposits is a frequent phenomenon that emerges in drying droplets with nonvolatile species [19,20,21,22]. The literature reveals that the coffee ring can appear in both simple and complex droplet systems [23,24,25,26]. The “coffee-ring effect” is a ring-like shape pattern observed in the evaporation of droplets containing suspensions [27], large colloids, nanoparticles [28], or individual molecules [29]. Its existence is due to capillary flows that, during the evaporation, emerge to transport components to the contact line to compensate the loss of volatile molecules at the edge of the droplet [30]. Desiccation crack, crystal, and aggregate patterns of different characteristics and sizes emerge from specific mass transport and aggregation mechanisms in a wide variety of droplet drying processes [31,32,33]. Although significant advances have been made in the understanding of pattern formation in both inorganic and organic colloids [21,34], the theory capable of predicting pattern formation from drying droplets of biological fluids is still incomplete.

The understanding of the pattern formation of protein dried drops has been a starting point for the improvement of the growing field of medical diagnosis by drop deposition. Protein deposit formation depends on control parameter such as the substrate temperature, relative humidity, the size and contact angle of the drop, and protein concentration [35,36,37,38,39]. The evaporation of water droplets containing proteins leaves behind uniform deposits with cracks and a coffee-ring structure [38,40]. The patterns generated by the evaporation droplets of model biological fluids (protein + water) are the results of the competition between gelation and desiccation kinetics [38]. Dry droplets formed from binary mixtures of proteins solution and water generate patterns with a coffee ring, cracks, and interesting small circular structures such as dendritic branches, crystal clusters, and disorder chains of crystals [41]. The formation of these aggregates is the result of protein–substrate and protein–protein interactions that involve interesting phenomena such as the Vroman effect [42].

The addition of salts in drops containing proteins leads to the growth of complex aggregates such as Chinese arrows, rosettes, scallops, dendrite shapes, and zigzag patterns [40,43,44,45,46,47]. Consequently, the increment in salt concentration affects the distribution of the aggregates, on the formation of patterns, by the ion absorption on macromolecules [48,49]. On the other hand, if external variables on drop deposition change such as relative humidity (RH) and substrate temperature, not only is the rate of evaporation modified but also the final morphology of the deposit. It has been shown that high RH slows the rate of evaporation on colloidal and protein solutions [29,49,50,51]. This results in the formation of more regular and symmetric crystals due to ions adding on positions where energy is minimum. By contrast, at low RH, the ions attach at random positions, resulting in amorphous crystal structures. The substrate temperature can strongly affect the modes of drying and the final pattern of colloidal and protein drops [24,52,53,54]. Indeed, the interesting pattern called “eye-like pattern”, a large aggregate enclosed in a uniform coating, results from a conformational change from native to irreversible folding of proteins induced by a hot substrate [54].

Although in the scientific literature, several works report the pattern formation of dried droplets of proteins [24,40,43,44,45,46,47,48,49,52,53,54,55], the effectiveness of this method to reveal the coexistence of macromolecules of the same species, with different conformational states, is still unknown. Advances in strategies to detect conformational changes in proteins through the analysis of deposits could lead to the development of rapid methodologies capable of diagnosing pathologies.

In order to evaluate the effectiveness of texture analysis in detecting macromolecules with different conformational states, in this paper, we report an experimental study on pattern formation of dried droplets of mixtures of bovine serum albumin (BSA) in NaCl solutions. We explored pattern formation in an open system (at fixed relative humidity), and in a closed system (where the relative humidity increases during the evaporation). We found that regardless of the type of system, at a low relative concentration of unfolded proteins $\phi_{u}$, small amorphous aggregates are formed. On the basis of the radial intensity profile, the mean pixel intensity, and entropy, we characterized and differentiated patterns formed at different relative concentrations of unfolded protein. Finally, we proved that it is possible to achieve 100% accuracy in identifying 4% of BSA contained in a binary mixture of proteins in saline solution.

## 2. Materials and Methods

### 2.1. Protein Solutions Preparation

Bovine serum albumin (BSA) powder (Sigma-Aldrich, A2153) and sodium chloride (NaCl) were used to prepare concentrated solutions of BSA and NaCl. These powders were dissolved, as received, in deionized (DI) water (Milli-Q, 18.2 MΩ cm) at 25 ${}^{\circ }$C at a concentration of 2.0 g/100 mL. The solutions were not further processed or purified. These concentrated solutions at $\phi_{p}$ = 2.00 wt% were diluted in NaCl solutons in variable amounts to create the concentrations: BSA ($\phi_{p}$ = 1 wt%) and NaCl ($\phi_{s}$ = 0.1 wt% and 1 wt%). The BSA unfolded solutions were derived from the native BSA solutions. These last were subjected to a water bath at 90 ${}^{\circ }$C for 10 min.

The protein solution with different relative concentration $\phi_{u}$ was prepared from the solutions of BSA in folded and unfolded states ($\phi_{p}$ = 1 wt%, and $\phi_{s}$ = 0.1 wt% and 1 wt%, respectively). The solutions were mixed according to the desired relative concentrations of unfolded protein $\phi_{u}$: 0%, 1%, 2%, 4%, 8%, 16%, 32%, 64%, and 100%. Therefore, $\phi_{u}$ = 0% and 100% means solutions without unfolded proteins and without folded proteins, respectively.

### 2.2. Droplet Evaporation

Nine drops of protein solution with a volume of 2 μL was deposited on clean substrates. The drops were dried under controlled temperature at T = 25 ${}^{\circ }$C. To evaluate the effectiveness of texture analysis to detect unfolded proteins in dried droplets formed with different control parameters, we studied droplet drying in two different systems. In the first (open system), ultraclean acrylic slides were used, placed in a 15 × 15 × 15 cm container with RH controlled at 30%. Here, the relative humidity was controlled using the effect of water activity $a_{w}=\rho /\rho_{0}$, where ρ is the vapor pressure of water in a substance and $\rho_{0}$ is the pressure of pure water vapor at the same temperature. The RH value was measured with a temperature and humidity sensor (Steren Ter-150). In the second system (closed system), we used ultrapure glass coverslips that were placed inside a 2 × 2 × 2 cm petri dish. Here, the relative humidity increase during the droplet evaporation. The initial relative humidity was 30%, reaching 70% at the end of the drying.

### 2.3. Image Acquisition

Pattern formation of dried droplets was observed using a Nikon camera (D3200) coupled with an optical microscope (VELAB-VE-M4). The lateral drying of the drops was recorded with a digital microscope at a resolution of 2 megapixels. The acquisition of images of the patterns in dry drops was carried out with a Nikon camera (D3300) coupled to a microscope (VELAB-VE-M4). The deposits were virtually divided into 4–6 quadrants. An image was taken at a resolution of 12 megapixels for each quadrant. Finally, the complete image of the dried droplet was reconstructed at a resolution of 6 megapixels.

### 2.4. Image Analysis

Texture analysis of the patterns in dried droplets was performed using the radial density profile I(r), the mean intensity of pixels, and entropy estimated from the gray level co-occurrence matrix (GLCM). Texture analysis of the patterns in dried droplets was performed using plugins of ImageJ software (radial profile, analyze, and GLCM texture) to estimate the radial density profile I(r), the mean intensity of pixels, and entropy estimated from the gray level co-occurrence matrix (GLCM). The mean intensity of pixels estimates the average mass contained in a region. The radial density profile describes a mass distribution in concentric circles as a function of radial distance, as follows:

(1) $$I\left(r\right)=\frac{1}{2\pi }\int_{0}^{2\pi }i\left(r,\theta \right)d\theta ,$$

where i(r,θ) is the local light intensity contained in a circle of radius *r*. Each point on the I(r) profile is the sum of the pixel intensities around a circle with radius *r* [16].

Entropy is defined from gray level co-occurence matrix. According to [56,57], we calculated the probability of variation between gray level *i* and *j* in a displacement distance (*d*) and angle (ϕ) with the matrix element p(i,j) given by:

(2) $$p\left(i,j\right)=\frac{C(i,j)}{\sum_{i=0}^{N_{g}-1}\sum_{j=0}^{N_{g}-1}C\left(i,j\right)}.$$

The number of events of gray levels *i* and *j* in the virtual measurement window of the (d,ϕ) pair is denoted by C(i,j). The value of p(i,j) is delimited by the size $N_{g}$×$N_{g}$, which is the upper limit.

The mean $u_{i}$ and the standard deviation $\sigma_{i}$, i=x,y, where *x* represent the columns and *y* the rows, were calculated with the following equations:

(3) $$u_{x}=\sum_{i=0}^{N_{g}-1}\sum_{j=0}^{N_{g}-1}i\cdot p\left(i,j\right),\;\sigma_{x}=\sum_{i=0}^{N_{g}-1}\sum_{j=0}^{N_{g}-1}{\left(i-u_{x}\right)}^{2}\cdot p\left(i,j\right),$$

(4) $$u_{y}=\sum_{i=0}^{N_{g}-1}\sum_{j=0}^{N_{g}-1}j\cdot p\left(i,j\right),\;\sigma_{y}=\sum_{i=0}^{N_{g}-1}\sum_{j=0}^{N_{g}-1}{\left(j-u_{y}\right)}^{2}\cdot p\left(i,j\right).$$

Entropy is as follows:

(5) $$H=-\sum_{i=0}^{N_{g}-1}\sum_{j=0}^{N_{g}-1}p\left(i,j\right)log\left(p\left(i,j\right)\right).$$

This quantity gives the heterogeneity of an image. Higher (lower) entropy values mean large (small) heterogeneous regions enclosed in an image.

Finally, we calculated ROC curves to differentiate among groups of deposits. Each ROC curve was calculated by comparing texture parameters of deposits formed at $\phi_{u}$ = 0% and metrics of deposits groups containing unfolded BSA (from $\phi_{u}$ = 1 to 100%). Each group of deposits was created by 18 deposits formed at specific $\phi_{p}$ and $\phi_{u}$. Therefore, the ROC curves were created by plotting the true positive rate (sensitivity) and the false positive rate (1-specificity). Under this scheme, the area under the ROC curves (AUC) provides information to evaluate the efficiency of classification algorithms between two groups. The closer the AUC to 1, the greater the probability of differentiating between groups. Detailed information on the calculation of ROC curves was recently presented by Carreón et al. [54].

## 3. Results

### 3.1. Patterns in Dried Droplets of Proteins

**Figure 1:**
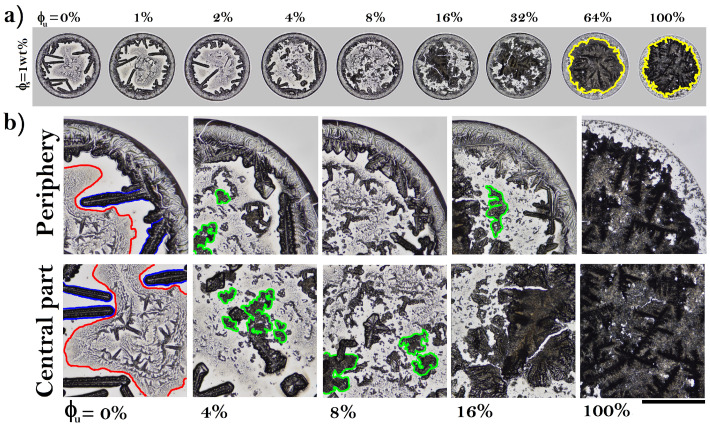
(**a**) Dried drops of protein mixtures produced at high concentration of NaCl ($\phi_{s}$ = 1 wt%). The corresponding deposit diameters are: 2.95, 2.87, 2.84, 2.91, 2.79, 2.87, 2.89, 2.88, and 2.93 mm, respectively. (**b**) Patterns formed in the periphery and the central part of the deposit. Needle-like crystals (blue), the region of complex aggregates (red), small dispersed amorphous aggregates (green), and the large aggregate of the eye pattern (yellow). The black line indicates 0.65 mm.

Figure 1a shows deposits formed at different relative concentrations of unfolded proteins $\phi_{u}$ and high concentration of NaCl ($\phi_{s}$ = 1 wt%). Deposits without unfolded proteins ($\phi_{u}$ = 0%) show a well-defined coffee-ring structure enclosing large needle-like crystals, see Figure 1b. The central region of the deposit exhibits a small scalloped pattern intertwine with tiny needle-like crystals, forming a region of complex aggregates. At low concentrations of unfolded proteins (from $\phi_{u}$ = 4%), the length of needle-like structures decrease and emerges small dispersed amorphous aggregates close to tiny scalloped crystals located in the center of deposit. From $\phi_{u}$ = 8%, the needle-like crystals disappear, and an increment of amorphous crystals concomitant with a decrease in scalloped structures begins. Finally, at high concentrations of unfolded protein ($\phi_{u}$ = 64%), the eye pattern emerges (a structure formed by a large aggregate surrounded by a uniform region).

**Figure 2:**
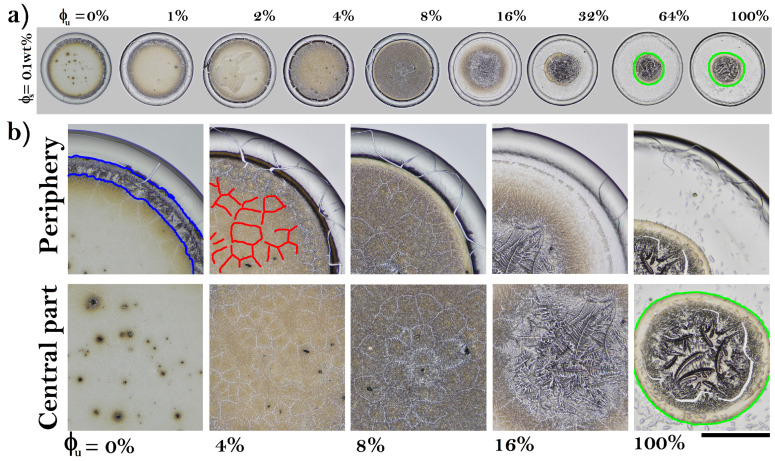
(**a**) Dried drops of folded and unfolded BSA containing low concentration of NaCl ($\phi_{s}$ = 0.1 wt%). The corresponding deposit diameters are: 2.58, 2.54, 2.56, 2.57, 2.56, 2.78, 2.95, 3.15, and 2.91 mm, respectively. (**b**) Patterns formed in the periphery and the central part of the deposit. The structure of interlaced crystals (blue), panels of dendritic crystals (red), and large interlocking crystals in the shape of a palm in the eye pattern (green). The black line indicates 0.65 mm.

**Figure 3:**
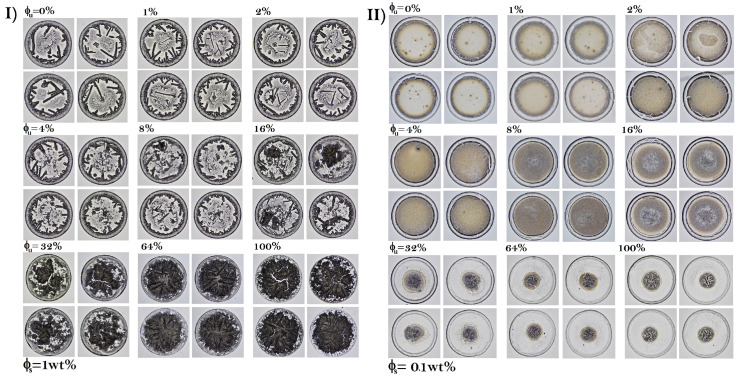
Reproducibility of patterns in dried drops of protein mixtures produced at (**I**) high and (**II**) low NaCl concentrations ($\phi_{s}$ = 1 wt% and 0.1 wt%, respectively). At $\phi_{s}$ = 1 wt% the corresponding average of deposit diameters are: 3.04 ± 0.22, 3.05 ± 0.18, 2.82 ± 0.16, 2.97 ± 0.12, 2.85 ± 0.19, 2.83 ± 0.28, 2.93 ± 0.17, 2.89 ± 0.15, and 2.97 ± 0.22 mm, respectively. At $\phi_{s}$ = 0.1 wt%, the corresponding average deposit diameters are: 2.56 ± 0.27, 2.52 ± 0.15, 2.55 ± 0.2, 2.59 ± 0.2, 2.59 ± 0.25, 2.81 ± 0.22, 3.03 ± 0.21, 3.2 ± 0.24, and 2.93 ± 0.28 mm, respectively.

Figure 2a shows dried droplets containing folded and unfolded BSA with a low concentration of NaCl ($\phi_{s}$ = 0.1 wt%). The coffee ring structure is clearly identifiable in all elements of this set of deposits. The dried droplets without unfolded proteins contain small-interlaced crystals that surround a set of panels formed by small well-defined dendritic regions, see Figure 2b. Some of these panels may contain a small aggregate. From $\phi_{u}$ = 4%, the dendritic structures in the panels are clearly denser, but the structure of interlaced crystals disappears. An eye pattern emerges at $\phi_{u}$ = 16%. The central aggregate is formed by a crystal pattern, resembling a palm leaf or fern frond, surrounded by small dendritic structures. From $\phi_{u}$ = 32%, the dendritic structures enclosing the eye pattern vanish while the aggregates that form the central pattern become wide. Figure 3 shows that, from a qualitative point of view, the deposit patterns of protein mixtures are highly reproducible.

**Figure 4:**
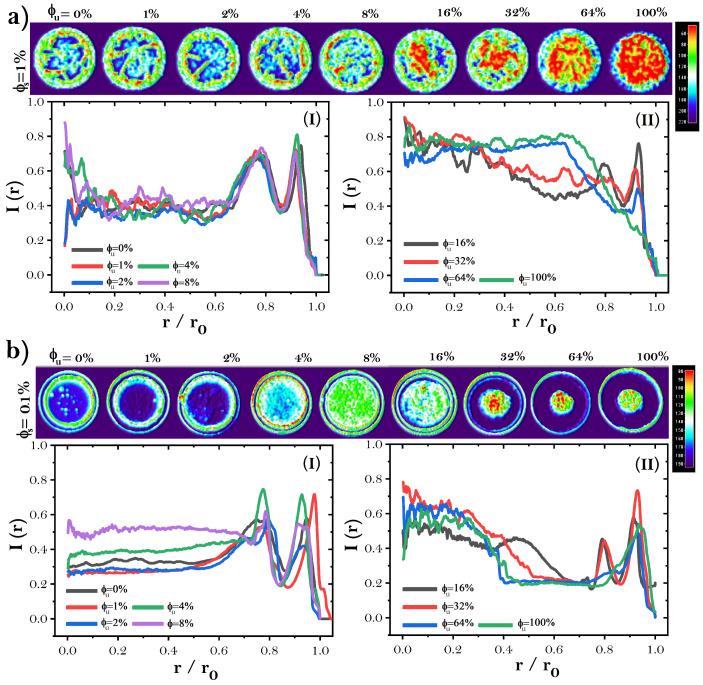
The 3D surface plots and the radial intensity profile I(r) of dried droplets of proteins at (**a**) high ($\phi_{s}$ = 1 wt%) and (**b**) low ($\phi_{s}$ = 0.1 wt%) NaCl concentration. (I) Patterns-like those formed without unfolded proteins. (II) Patterns with structural differences from dried droplets without unfolded proteins.

We use a 3D surface plot and radial intensity profile I(r) to quantitatively investigate the mass distribution in the protein deposits, see Figure 4. We found that dried droplets of mixtures of proteins formed at high NaCl concentration can be divided into two deposit groups: patterns structurally indistinguishable from deposits without unfolded proteins and patterns with different structural characteristics from the first group (unfolded proteins <16%). Figure 4a shows that, at low concentrations of unfolded BSA, from $\phi_{u}$ = 0 to 8%, the blue regions in the three-dimensional graph and the small picks curves of I(r) correlate with a low mass distribution in the central region of the deposits. The small red regions and the prominent peak of the I(r) (Panel I) show that mass distribution on the coffee ring is highest. In contrast, from $\phi_{u}$ = 16% (second group), the three-dimensional graph of protein deposits shows a big red region in the central part of the deposit that indicates the formation of the eye pattern of proteins. The corresponding I(r) (Panel II) shows that while the coffee ring vanishes, the mass in the central aggregate increases as a function of the relative concentration of unfolded BSA.

Dried droplets of proteins formed at low NaCl concentrations also can be classified in two deposit groups, (see Figure 4b). At a low concentration of unfolded BSA ($\phi_{u}$), for values from 0 to <4%, the blue spot in the three-dimensional graph indicates a low mass in the central region of deposits. The flat curves and the two large picks of the corresponding I(r) (Panel I) indicate a greater mass distribution in the coffee ring stain. The 3D surface plot and I(r) of deposits formed at $\phi_{u}$ = 8% reveal that there are no significant differences between the mass contained in the central region of the deposits and the coffee ring. Here, the big green region in the three-dimensional graph appears due to the high density of dendritic structures. The second group is formed by different pattern characteristics due to the increase in unfolded proteins. In this group, the blue spot observed in the previous group disappears. It can be observed that in deposits formed at $\phi_{u}$ = 16%, the 3D surfaces plot and the I(r) show some differences with the mass contained in the central region. Here, the high density of dendritic structures observed at 8% begins to disappear (green spots) to form the eye pattern. Finally, for values of unfolded BSA > 16%, the blue region shapes and the profile of the I(r) (Panel II) curves indicate the formation of the eye pattern.

### 3.2. Accuracy to Identify Unfolded Proteins

**Figure 5:**
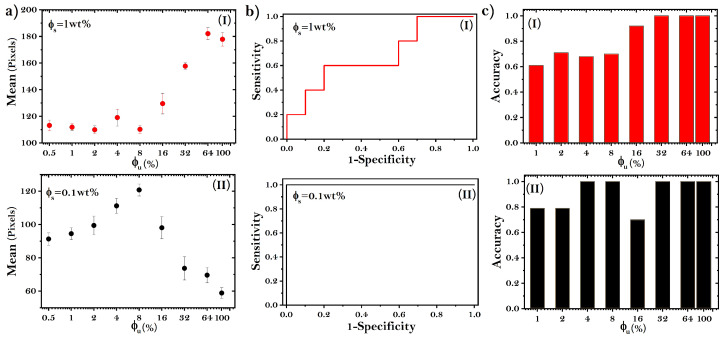
(**a**) The mean pixel intensity corresponding to dried droplets formed at $\phi_{s}$ = 1 wt% (Panel I) and 0.1 wt% (Panel II). (**b**) ROC curves plotted from the mean pixel intensity analysis of deposits produced at $\phi_{u}$ = 4%, and f$\phi_{u}$ = 1 (Panel I) and 0.1 (Panel II). (**c**) Accuracy for identifying deposit groups containing different concentrations of unfolded BSA. Panel I: corresponds to deposits formed at $\phi_{s}$ = 1 wt%, while Panel II represents the accuracy for deposits produced at $\phi_{s}$ = 0.1 wt%.

First, we use the mean pixel intensity to characterize the dried droplets, see Figure 5a. At high NaCl concentrations, the magnitude of the error bars indicates that there are no significant differences between patterns formed from $\phi_{u}$ = 0–8% (see Panel I in Figure 5a). However, from $\phi_{u}$ = 16%, the mean pixel intensity increases as a function of $\phi_{u}$. On the other hand, at low NaCl concentrations, the mean values increase to reach a maximum value at $\phi_{u}$ = 8% (see Panel II in Figure 5a). Interestingly, afterward, the values decrease monotonically as a function of $\phi_{u}$. Therefore, although at first glance the patterns in deposits formed at $\phi_{u}$ = 0 and $\phi_{u}$ = 16% are clearly different, this increasing–decreasing behavior in the mean pixel intensity produces similar values for these two groups of deposits.

In order to analyze the capability of the texture analysis on dried droplets to identify the minimum concentration of unfolded proteins in a solution, we used the receiver operating characteristic (ROC). Panels I and II in Figure 5b show ROC curves estimates from the mean pixel intensity values for $\phi_{u}$ = 4% at $\phi_{s}$ = 1 wt% and 0.1 wt%, respectively. The monotonically increasing behavior of the ROC curve means a poor differentiation between groups (Panel I in Figure 5b). By contrast, the step shape of the corresponding ROC curve at low NaCl concentrations indicates a good differentiation between groups of deposits (Panel II in Figure 5b). To determine the optimal sensibilities and specificities, we carried out the sum of sensitivity and specificity considering specificity values larger than 50%. Thereafter, the values with the highest sensitivity were selected. The optimal sensibilities and specificities from these the ROC data are 0.6, 0.6; and 0.8, 1, respectively.

The area under the ROC curve (AUC) gives the probability that a dried droplet produced by mixtures of folded and unfolded BSA can be classified as a deposit without unfolded proteins. Panel I in Figure 5c shows the accuracy of the mean pixel intensity to classify protein solutions with high NaCl concentrations. From $\phi_{u}$ = 1–8%, the accuracy of detecting unfolded BSA is below 70%. However, the values increase in the upper levels, achieving 93% accuracy in identifying deposits with 16% unfolded proteins. The accuracy of detecting unfolded BSA improves at low NaCl concentrations (see Panel II in Figure 5c). Here, the texture analysis based on the mean pixel intensity achieves 100% accuracy in identifying dried droplets with 4% unfolded BSA. Note that although at the naked eye the patterns formed without unfolded proteins and those produced with 16% unfolded proteins are clearly different, the accuracy in the differentiation of these two groups of deposits is less than 70%. This occurs because the values of mean pixel intensity are quite similar.

**Figure 6:**
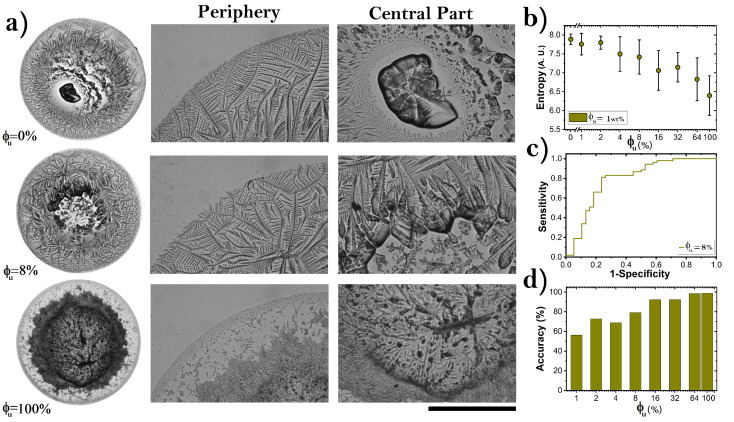
(**a**) Dried droplets of proteins produced in a small acrylic box ($\phi_{s}$ = 1 wt%, and $\phi_{u}$ = 0%, 8%, and 100%. The initial and the final relative humidity were 30% and 60%, respectively. The corresponding deposit diameters are: 6.3, 4.7, and 4.5 mm, respectively. (**b**) Entropy values of dried droplets produced at different $\phi_{u}$ in confined conditions. (**c**) ROC curve plotted from the entropy evaluation of deposits produced at $\phi_{u}$ = 8%. (**d**) Accuracy for identifying unfolded BSA in different groups of deposits. The black line indicates 1 mm.

To assess the potential use of texture analysis as a tool capable of detecting the low concentrations of unfolded proteins in deposits produced at different drop drying conditions, we analyzed dried droplets of proteins formed on a glass substrate placed inside a small acrylic box (system closed, for details, see Materials and Methods). Under these confinement conditions, the relative humidity increase from 30% to 70% while the drying of the drops occurs. Figure 6a show patterns in dried droplets of proteins formed at $\phi_{u}$ = 0%, 8%, and 100%, at $\phi_{s}$ = 1 wt%. The first two deposits are characterized by a palm leave or fern frond morphology at the periphery of the dry drop. The most obvious morphological difference between the two is observed in the central region. Deposits without denatured proteins show a large crystalline aggregate surrounded by dendritic pattern and small crystals. By contrast, deposits with $\phi_{u}$ = 8% show only small dispersed amorphous aggregates. At high concentrations of $\phi_{u}$, the eye pattern is clearly observed.

We measure the entropy of the deposits to prove that it is possible to use any other texture parameter to carry out a characterization and detection of unfolded proteins in dried droplets. Figure 6b shows that entropy values decrease as a function of the $\phi_{u}$. Clearly, there are no significant differences between the entropy values of $\phi_{u}$ = 0–8%. The quasistep shape of the ROC curve calculated from the entropy values for $\phi_{u}$ = 8% indicates a regular differentiation between groups of deposits, see Figure 6c. Figure 6d shows that, from $\phi_{u}$ = 16%, the accuracy of the entropy to detect unfolded BSA reaches satisfactory values (higher than 90%).

### 3.3. Pattern Formation in Dried Droplets of Folded and Unfolded Proteins

**Figure 7:**
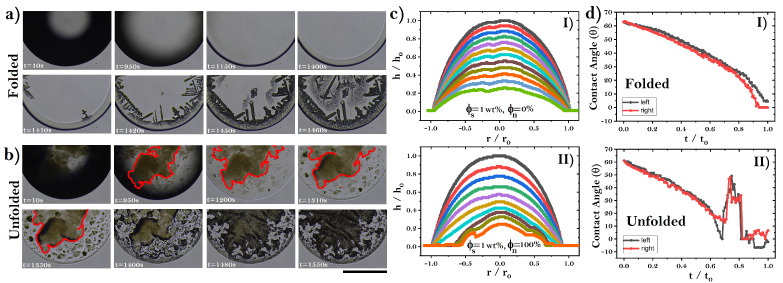
(**a**) Image sequences on the pattern formation of dried droplets of folding proteins on acrylic substrate ($\phi_{s}$ = 1 wt% and $\phi_{u}$ = 0). (**b**) Pattern formation in droplets of unfolded BSA on acrylic substrate ($\phi_{s}$ = 1 wt% and $\phi_{u}$ = 100%). The red regions show aggregates suspended in the center of the droplet. The black line indicates 1 mm. (**c**) The corresponding lateral profile of droplets in Panels I and Panel II of (**a**). (I) $t/t_{0}$ = 0, 0.06, 0.13, 0.19, 0.25, 0.32, 0.38, 0.44, 0.51, 0.57, 0.64, and 0.71, respectively. II) $t/t_{0}$ = 0, 0.1, 0.2, 0.31, 0.41, 0.51, 0.61, 0.72, 0.82, and 0.91, respectively, and (**d**) the evolution of the contact angle of droplets in (**a**,**b**).

To examine how unfolded proteins give rise to amorphous aggregates, we studied the pattern formation from the evaporation of protein droplets. Figure 7a shows an image sequence of typical pattern formation from the drying of native protein droplets. During the process, capillary flows draw large numbers of proteins to the edge, forming the well-known coffee ring structure (see *t* = 1400 s in Figure 7a). Then, once the salt saturation concentration is reached, needle-like crystals pointing inward emerge from the coffee ring (*t* = 1410 and 1420 s). Later, small scalloped and tiny needle-like crystals are interlaced in the central region to form complex patterns. On the other hand, droplets of unfolded proteins form aggregates suspended mostly in the center of the droplet (see red regions in Figure 7b). As a result, amorphous crystallization emerges in the center of the dry drop to form the eye pattern. The interaction of unfolded proteins of BSA gives rise to aggregates of proteins through the association of lateral chains by a reaction of sulfhydryl and disulfide groups [54,58]. During the entire drying process, the suspended protein aggregates remain without relative movement (from *t* = 10 to 1350 s). The protein aggregates are then precipitated onto the substrate. Crystal growth occurs on amorphous protein crystallization. The drop lateral profile shows that, in the last evaporation stages, significant differences arise in the drying process between both drops; see Panels I and II in Figure 7c. In the gel phase of folded protein droplets, the fluid contracts anisotropically, from the contact line to the center of the droplet. The behavior of the contact angle is in accordance with this phenomenon; see Panels I and II in Figure 7d.

## 4. Discussion

The detection of conformational states of macromolecules is of great relevance for the diagnosis of pathologies such as Huntington’s disease, Parkinson’s disease, prion diseases, and Alzheimer’s disease [1,3,7]. Currently, texture analysis of dried droplet deposits of bio-fluids has contributed to the detection of health problems [12,14,15]. However, it is unknown whether, through the analysis of the texture of dry droplets, it is possible to detect conformational changes in macromolecules. The development of a protocol through which texture analysis of dried droplets can detect conformational changes would facilitate the development of rapid tests and may improve currently available diagnostic tests.

In this study, we carried out texture analysis on patterns in dried droplets to identify unfolded BSA. We studied droplet drying in two different systems. In the first one (open system), the drops were placed on an acrylic slide and evaporated at fixed relative humidity. In the second one (closed system), the drops were placed on a glass cover slip confined within a small box, where the relative humidity increased with the evaporation of the drops. We show that by using texture analysis on dried droplets, it is possible to achieve high accuracy in identifying low relative concentrations of BSA in the unfolded conformational state. The eye pattern is the most distinctive feature of unfolded protein deposits. This pattern is composed of amorphous crystals of NaCl and aggregates located in the center of the deposit, which are surrounded by a smooth zone characterized by the absence of crystals. A previous study demonstrated that the formation of eye pattern in protein films on a hot substrate (above 58${}^{\circ }$) correlates with the lateral association by a chain reaction of sulfhydryl and disulfide groups that give rise to processes of molecular aggregation [54]. This report is consistent with our finding that a major concentration of unfolded BSA results in significant molecular aggregation, generating an eye pattern formation.

Some previous studies demonstrated that NaCl induces the formation of complex aggregates in alcoholic drinks and medicines [56,59]. Our finding show that NaCl generates complex aggregates that allow one to classify and differentiate among deposit groups. Therefore, based on these data, we propose that NaCl is fundamental to facilitating the detection of unfolded BSA through the morphological analysis of patterns in dried droplets. Interestingly, the efficiency in the detection of unfolded BSA is at its maximum at low NaCl concentrations.

Texture analysis of dried droplets has some inherent advantages over existing methods to detect conformational changes in BSA. This analysis is easy to perform, is fast, and does not require sophisticated instrumentation and highly qualified personnel. Notably, this method can achieve high efficiency in detecting folded and unfolded states of BSA. The results presented above are an advance in the development of methods to detect proteins irreversibly unfolded and represent an advance in the knowledge of the formation of dried droplets of proteins. However, the method presented here has some limitations.

Analysis of the texture of the dry droplets does not provide new information on the structural state of the unfolded BSA. In this sense, this method is limited only to detecting the concentrations of unfolded BSA in solution. Indeed, the aggregation of this protein results in amorphous aggregates in the dry droplets. Therefore, proteins with different aggregation processes compared to BSA or proteins that do not involve lateral association by a chain reaction of sulfhydryl and disulfide groups may have different morphological characteristics.Therefore, this method can be considered as a rapid test that only determines the existence of irreversible structural changes in BSA or similar proteins. Another disadvantage of performing unfolded protein detection using a single physical morphological observable is that it could produce false positives and false negatives. An example of this is the classification of deposits with 16% folded proteins. From Figure 3, at first glance, these dried droplet patterns are different from those produced purely with unfolded proteins. However, texture analysis based on average pixels cannot differentiate between the two groups.

Partially unfolded proteins, salts, relative humidity, type of substrate, and initial droplet volume drive the aggregation and mass transport mechanism that give rise to the final morphology of patterns in dried droplets [40,43,44,47,48,49,52,54]. Therefore, exploring how these control parameters modify the efficiency of the detection of proteins in different conformational states is an investigation that we would like to do in the future.

In conclusion, we present an experimental study on patterns formed by the evaporation of droplets of sodium chloride (NaCl) and bovine serum albumin (BSA), in folded and unfolded states. Regardless of the NaCl concentration, the type of substrate, and relative humidity, the formation of small amorphous aggregates reveals the presence of low unfolded protein concentrations. Moreover, at a high relative concentration of unfolded BSA above 16%), the “eye-like pattern” emerges. The radial intensity profile, the mean pixel intensity, and entropy allow for characterizing and differentiating protein deposits.

Overall, the texture analysis of patterns in dried droplets can detect, with 100% accuracy, a relative concentration of 4% BSA in the unfolded conformational state. This finding could have potential use in detecting structural changes in macromolecules associated with relevant pathologies.

## Data Availability

Y.J.P.C. wish to acknowledge financial support from DGAPA-UNAM. For technical support, we thank ECOn BG. The authors appreciate the reviewers for their helpful comments and suggestions in this study.
